# Montelukast treats *Streptococcus pneumoniae*-induced sepsis via antibacterial and anti-inflammatory activities

**DOI:** 10.1128/spectrum.01221-25

**Published:** 2025-10-15

**Authors:** Wei Cao, Dongjun Xu, Huijie Yu, Xuning Shen

**Affiliations:** 1 Emergency Medicine Department, Affiliated Hospital of Jiaxing University, Jiaxing University 417382, Jiaxing, Zhejiang, China; Petrified Bugs LLC, Miami, Florida, USA

**Keywords:** *S. pneumoniae*, montelukast, antibacterial, anti-inflammatory, sepsis

## Abstract

*Streptococcus pneumoniae* is a leading causative pathogen of community-acquired pneumonia and sepsis. The increasing prevalence of antibiotic resistance among *S. pneumoniae* strains poses a major challenge to conventional antibiotic therapies. This study aimed to systematically evaluate the antibacterial, anti-biofilm, and *in vivo* therapeutic effects of montelukast against *S. pneumoniae* and to explore its potential for drug repurposing as an anti-infective agent. *In vitro* susceptibility testing revealed that montelukast exhibited inhibitory activity against both standard reference strains and 11 clinical multidrug-resistant (MDR) *S. pneumoniae* isolates, with minimum inhibitory concentrations (MICs) ranging from 4 to 32 µg/mL. Time-kill assays using the representative MDR strain S12 showed that montelukast at 16 µg/mL could completely eradicate bacteria within 8 h. And biofilm assays further demonstrated that montelukast at concentrations of 8–16 µg/mL significantly disrupted preformed biofilms and reduced viable bacterial counts within mature biofilms, suggesting its potential to combat chronic infections. In a mouse model of sepsis, montelukast treatment significantly improved survival rates of infected mice, with the 10 mg/kg dosage group achieving a 7-day survival rate of 80%. Quantification of bacterial burden revealed a marked reduction in colony-forming units (CFUs) in both blood and lung of montelukast-treated mice. Furthermore, analysis of serum inflammatory cytokines indicated that montelukast effectively suppressed the secretion of pro-inflammatory mediators such as TNF-α and IL-6, suggesting a dual role in infection control and inflammation modulation. In conclusion, montelukast exhibits potent anti-*S*. *pneumoniae* activity, including bactericidal effects against resistant strains, biofilm disruption capabilities, and synergistic antibacterial and anti-inflammatory effects *in vivo*. These findings support the potential repurposing of montelukast as a therapeutic agent for *S. pneumoniae* infections, particularly sepsis.

**IMPORTANCE:**

Montelukast showed promise as a repurposed treatment for multidrug-resistant *Streptococcus pneumoniae*, especially in severe infections like sepsis. It demonstrated strong *in vitro* activity against both sensitive and resistant strains and exerted concentration-dependent bactericidal effects and cleared biofilms. In a mouse sepsis model, montelukast improved survival, reduced bacterial load, and mitigated inflammation. Mechanistically, it disrupted membrane integrity and induced oxidative stress and may target pseudouridine synthase. These findings supported montelukast’s potential as an antimicrobial with dual antibacterial and anti-inflammatory action.

## INTRODUCTION

*Streptococcus pneumoniae* (*S. pneumoniae*) is a Gram-positive, non-spore-forming, spherical bacterium that typically appears in pairs or short chains (1). It is one of the most common human pathogens and is frequently carried in the upper respiratory tract, particularly in infants, the elderly, and immunocompromised individuals (1, 2). Due to its high pathogenicity and airborne transmission capability, *S. pneumoniae* can cause a wide range of infections. These infections often lead to severe complications in individuals with impaired immune function. The diseases caused by *S. pneumoniae* are associated with high morbidity and mortality rates, posing a significant global public health threat (3). The most common disease caused by *S. pneumoniae* is pneumonia, particularly community-acquired pneumonia (CAP) (3). According to a large retrospective study conducted, *S. pneumoniae* was responsible for approximately 66% of CAP cases (2). In addition, it can also cause meningitis, acute otitis media, sinusitis, and septicemia. Among these, sepsis caused by *S. pneumoniae*—a systemic inflammatory response syndrome—has attracted significant research attention due to its rapid progression and high mortality rate (4). Sepsis is a life-threatening condition triggered by infection, characterized by sustained immune activation and widespread organ dysfunction (5, 6). *S. pneumoniae*-induced sepsis represents a common and fatal clinical manifestation (3, 7). Patients with sepsis often present with hypotension and multiple organ failure, and the rising resistance to standard antibiotics has further complicated treatment.

The pathogenesis of *S. pneumoniae*-induced sepsis is complex. Studies have shown that the bacteria can activate the host immune response via its surface polysaccharides and other cell wall components, leading to the overproduction of pro-inflammatory cytokines such as tumor necrosis factor-α (TNF-α) and interleukin-6 (IL-6), thereby inducing systemic inflammation (8–10). This dysregulated immune response, driven by excessive release of inflammatory mediators, contributes to increased vascular permeability, organ damage, and ultimately the development of sepsis (11). Furthermore, *S. pneumoniae* can evade immune surveillance by forming biofilms, enhancing its persistence and resistance during infection (12). Clinically, the diagnosis and treatment of *S. pneumoniae*-induced sepsis remain challenging. The early symptoms often resemble those of other infections, leading to diagnostic delays. Current treatment relies heavily on antibiotic therapy and supportive care, but the increasing prevalence of resistant strains has gradually undermined therapeutic efficacy (13–15). Sepsis-associated *S. pneumoniae* isolates showed varying degrees of resistance to commonly used antibiotics, such as penicillin and erythromycin (16, 17). The emergence of resistant strains has made the management of severe complications such as sepsis more difficult. Existing antimicrobial therapies face significant limitations, underscoring the urgent need to develop novel antibiotics or therapeutic strategies, particularly those effective against multidrug-resistant (MDR) *S. pneumoniae* (13, 18). In summary, *S. pneumoniae* is a major pathogen that causes severe diseases, including sepsis, and represents a significant global health concern. The increasing emergence of drug-resistant strains necessitates further research and innovation in infection prevention and treatment strategies.

Traditional antimicrobial treatments for *S. pneumoniae* infections primarily rely on β-lactam antibiotics (such as penicillins and cephalosporins) and macrolides (19). However, the rising prevalence of resistant strains has made treatment increasingly challenging. Consequently, innovative antimicrobial strategies, especially drug repurposing, have become an important focus in current therapeutic research (20). Drug repurposing involves re-evaluating the antimicrobial properties of approved drugs, particularly those originally indicated for non-infectious diseases (21). These drugs often have well-established safety profiles and pharmacokinetic data, allowing for faster clinical translation while bypassing many of the safety and efficacy hurdles typically encountered in new drug development (22). Additionally, drug repurposing can offer cost-effective treatment alternatives and expand therapeutic options amid rising antibiotic resistance (23). Notably, some repurposed drugs exhibit potent antibacterial activity against specific pathogens and may help address the growing issue of antimicrobial resistance (24). Montelukast, a leukotriene receptor antagonist commonly used to treat allergic conditions and asthma, has recently gained attention for its potential antibacterial properties (25, 26). While montelukast’s primary mechanism is to alleviate allergic symptoms by inhibiting leukotriene receptors, emerging studies have shown that it can inhibit biofilm formation in *Pseudomonas aeruginosa* and suppress the growth of certain Gram-positive bacteria (27–29). However, limited research has explored the therapeutic potential of montelukast against *S. pneumoniae*, particularly in the context of sepsis, highlighting the need for further investigation into its antimicrobial mechanisms and clinical applicability. This study aims to investigate the therapeutic effects of montelukast in *S. pneumoniae*-induced sepsis, with a particular focus on its antibacterial efficacy and underlying mechanisms. We evaluated the bactericidal activity of montelukast through both *in vitro* and *in vivo* models, including a mouse sepsis model, to assess its impact on clinical symptoms, organ function, and survival outcomes. This work aims to provide a novel therapeutic approach for the treatment of *S. pneumoniae* infections, especially in the face of increasing multidrug resistance.

## RESULTS

### Bactericidal activity of montelukast against multidrug-resistant *S. pneumoniae*

**Fig 1:**
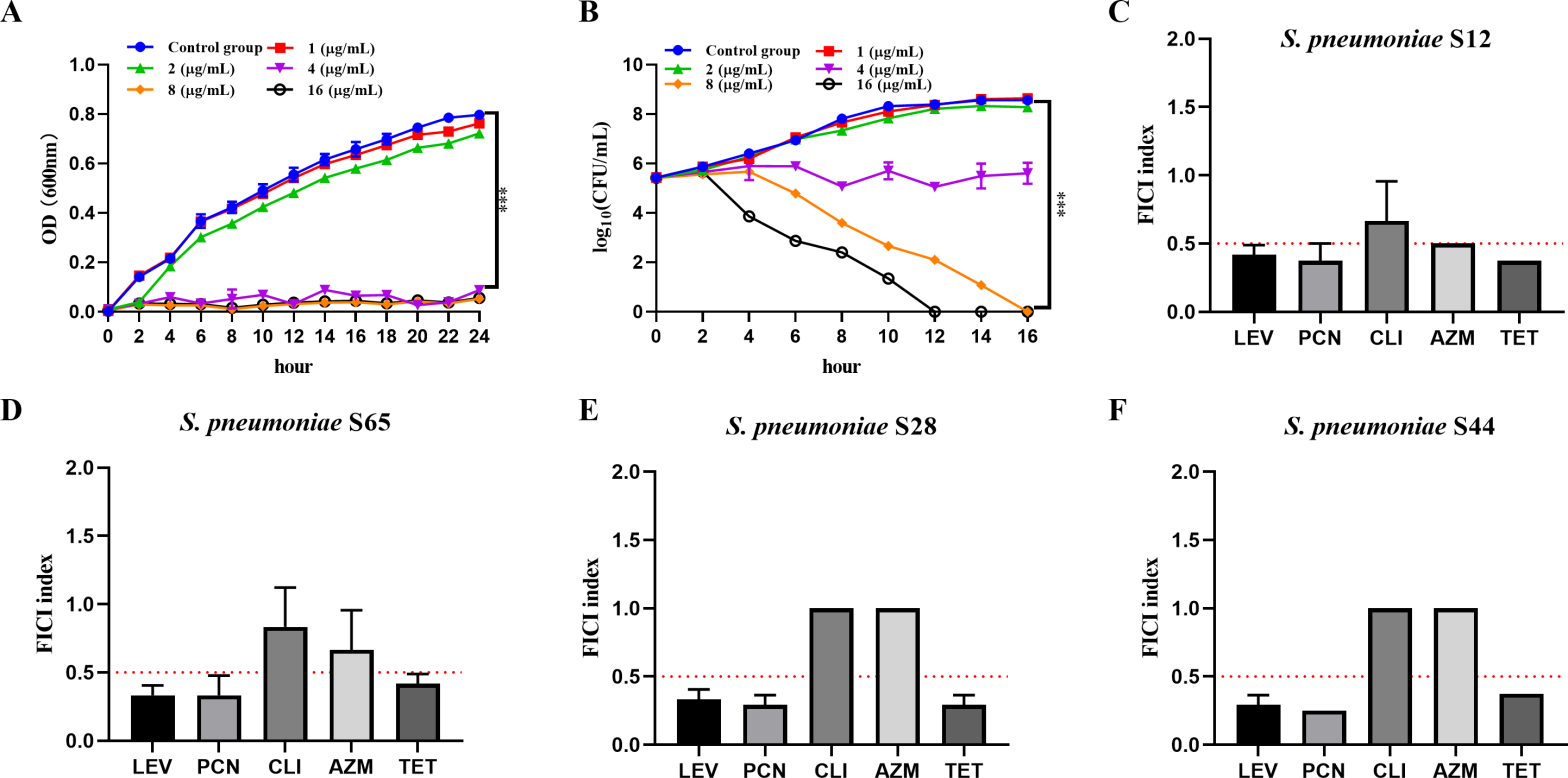
Evaluation of montelukast-mediated growth inhibition and bactericidal activity against *S. pneumoniae* strain S12. (**A**) Growth curves of S12 treated with montelukast at various concentrations (1, 2, 4, 8, 16 µg/mL), assessed by OD600 measurements. (**B**) Bactericidal activity assessed by CFU enumeration after treatment with montelukast. Synergistic antibacterial effects of montelukast sodium in combination with different antibiotics against four clinical isolates of *S. pneumoniae* (S12 [**C**], S65 [**D**], S28 [**E**], and S44 [**F**]), evaluated by the fractional inhibitory concentration index (FICI). Montelukast was combined with levofloxacin (LEV), penicillin (PCN), clindamycin (CLI), azithromycin (AZM), or tetracycline (TET). The dotted red line indicates the threshold of FICI = 0.5, below which synergistic interaction was defined. Experiments were performed in biological triplicates. Data are presented as mean ± standard deviation (Mean ± SD) (*n* = 3). Statistical analysis was performed using unpaired two-tailed *t*-test (***P < 0.001).

In this study, we first conducted *in vitro* antimicrobial susceptibility testing of montelukast and commonly used antibiotics against standard strains (*S. pneumoniae* NCTC 7466 and ATCC 49619) and 11 clinical isolates preserved in our laboratory from Zhejiang Province, China (S12, S65, S63, S42, S28, S23, S44, S32, S13, S17). The results showed that the minimum inhibitory concentration (MIC) values of montelukast against all strains ranged from 4 to 32 µg/mL, with the majority exhibiting MICs of 4 µg/mL, indicating a certain level of *in vitro* antibacterial activity against *S. pneumoniae* (Table 1). In contrast, the tested strains displayed varying degrees of resistance to conventional antibiotics such as levofloxacin (LEV), penicillin (PCN), clindamycin (CLI), azithromycin (AZM), and tetracycline (TET). Notably, several strains, including S65, S42, and S23, exhibited high MICs against PCN (16–32 µg/mL), suggesting the presence of multidrug resistance (Table 1). Although vancomycin (VAN) and linezolid (LIN) retained good activity against these clinical isolates (MICs < 2 µg/mL), a few strains showed elevated MICs up to 8 µg/mL (Table 1). Collectively, these findings indicate a widespread trend of multidrug resistance among clinical isolates of *S. pneumoniae*. Importantly, montelukast exhibited comparable *in vitro* activity against both susceptible and resistant strains, providing a rationale for further investigation into its dual antibacterial and anti-inflammatory therapeutic potential for *S. pneumoniae*-induced sepsis. To further evaluate the antibacterial activity of montelukast against multidrug-resistant *S. pneumoniae*, we selected strain S12, which demonstrated broad-spectrum resistance in preliminary tests, for dynamic assessment of bacterial growth and killing at various drug concentrations. As shown in Fig. 1A, bacterial growth inhibition was monitored by measuring optical density at 600 nm (OD600) after treatment with different concentrations of montelukast (1, 2, 4, 8, and 16 µg/mL). The results showed a dose-dependent inhibition of bacterial growth. In the control and 1 µg/mL groups, bacterial growth progressed rapidly, with OD600 values reaching approximately 0.8 within 24 h. The 2 µg/mL group showed a slightly slower growth rate, indicating partial inhibition. However, at concentrations of 4 µg/mL and above, bacterial growth was almost completely suppressed, with OD600 remaining at very low levels throughout the incubation period, consistent with the MIC results (Fig. 1A). Further evaluation of bactericidal activity via colony-forming unit (CFU) counting revealed that montelukast at 4 µg/mL only inhibited growth without significantly reducing bacterial counts over time (Fig. 1B). In contrast, treatment with 8 µg/mL and 16 µg/mL resulted in a marked, time-dependent decrease in CFU, with complete eradication of bacteria observed within 8 h at 16 µg/mL (Fig. 1B). To further explore the potential of montelukast sodium against *Streptococcus pneumoniae*, synergistic antimicrobial effects were evaluated. Montelukast combined with levofloxacin (LEV), penicillin (PCN), and tetracycline (TET) showed consistent synergy (FICI < 0.5) across all strains, particularly in S28 and S44. Combinations with clindamycin (CLI) and azithromycin (AZM) showed limited or strain-dependent synergy, with most FICI values near or above 0.5 (Fig. 1C through F). These results confirm that montelukast exhibits a concentration-dependent bactericidal effect against multidrug-resistant *S. pneumoniae* S12, particularly at concentrations ≥ 8 µg/mL, supporting its potential as a repurposed anti-infective agent.

**TABLE 1:** MICs of drugs for strain used in the study^*a*^

Strains	Source	MIC (µg/mL)							
		Montelukast	LEV	PCN	CLI	AZM	TET	VAN	LIN
*S. pneumoniae* NCTC 7466 (D39)		4	0.25	0.5	0.125	0.25	0.5	0.125	0.25
*S. pneumoniae* ATCC 49619		4	0.5	0.25	0.25	0.125	1	0.25	0.5
*S. pneumoniae* S12	China (Zhe Jiang)	4	4	16	32	8	32	8	8
*S. pneumoniae* S65	China (Zhe Jiang)	8	2	32	16	16	32	4	2
*S. pneumoniae* S63	China (Zhe Jiang)	8	4	8	16	4	32	8	1
*S. pneumoniae* S42	China (Zhe Jiang)	4	1	4	32	16	8	4	0.5
*S. pneumoniae* S28	China (Zhe Jiang)	16	4	2	8	32	16	8	2
*S. pneumoniae* S23	China (Zhe Jiang)	8	2	8	16	8	32	8	4
*S. pneumoniae* S44	China (Zhe Jiang)	16	2	16	8	16	8	4	4
*S. pneumoniae* S32	China (Zhe Jiang)	16	4	4	16	8	32	4	2
*S. pneumoniae* S13	China (Zhe Jiang)	4	4	4	4	8	4	0.5	1
*S. pneumoniae S*17	China (Zhe Jiang)	32	1	8	16	32	16	2	1

^*a*^ LEV, Levofloxacin; PCN, Penicillin; CLI, Clindamycin; AZM, Azithromycin; TET, Tetracycline; VAN, Vancomycin; LIN, Linezolid.

### Montelukast exhibited potent anti-biofilm activity against multidrug-resistant *S. pneumoniae*

**Fig 2:**
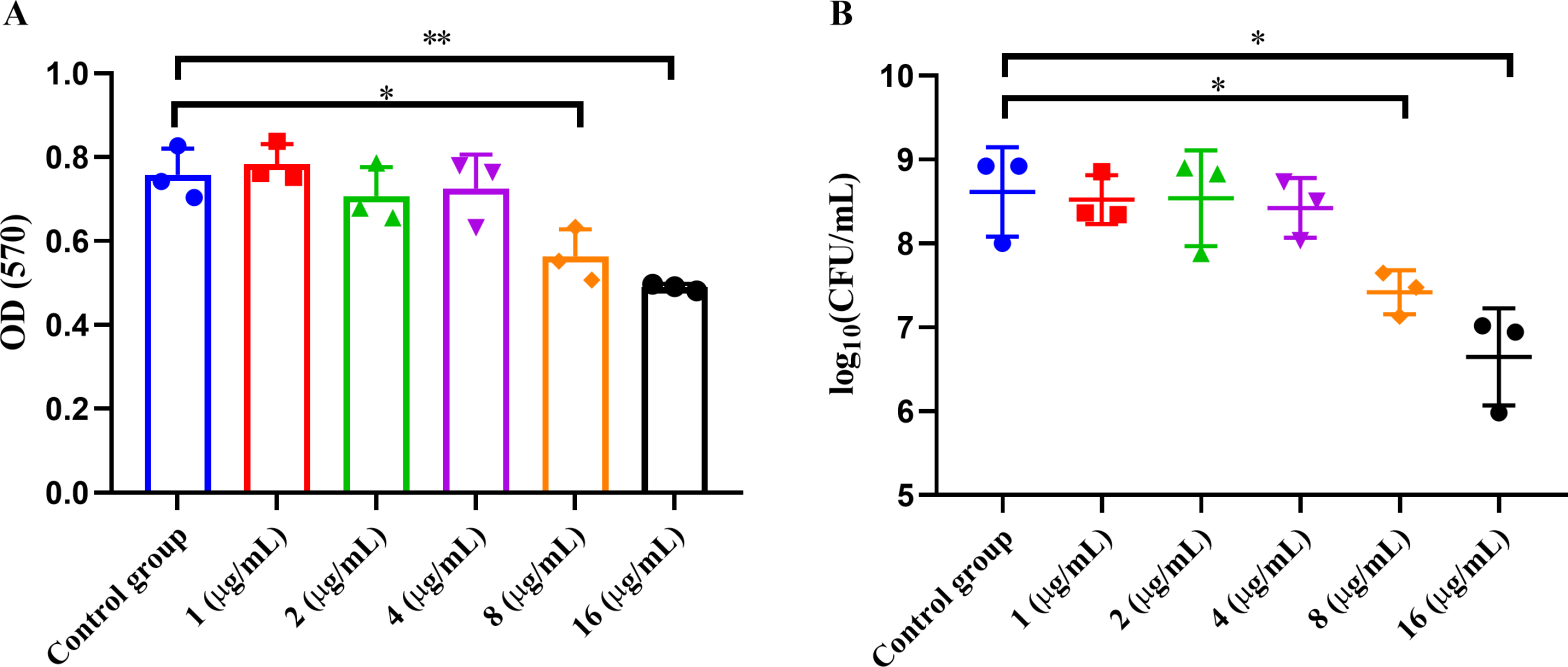
Evaluation of montelukast-mediated biofilm disruption activity in *S. pneumoniae* strain S12. (**A**) Crystal violet staining was used to quantify biofilm biomass after treatment with montelukast at 1, 2, 4, 8, and 16 µg/mL (OD570 as readout). (**B**) CFU enumeration of viable bacteria within biofilms following montelukast treatment. Bacteria and drug were co-incubated for 24 h at 37°C. Experiments were performed in biological triplicates. Data are presented as Mean ± SD, and statistical analysis was performed using unpaired two-tailed *t*-test (*P < 0.05, **P < 0.01).

*S. pneumoniae* often forms biofilms in chronic and recurrent infections, which enhance bacterial adhesion and protection, reduce antibiotic penetration, and impair immune clearance, which contributes significantly to treatment failure and the emergence of antibiotic resistance (30, 31). Therefore, effective disruption of biofilms is critical for improving infection control. In this study, we further evaluated the anti-biofilm effects of montelukast against the multidrug-resistant strain S12. Biofilm biomass was assessed by crystal violet staining and quantified using OD570 measurements. The results showed no significant reduction in biofilm biomass at low concentrations of montelukast (1–4 µg/mL) (Fig. 2A). However, treatment with 8 µg/mL and 16 µg/mL led to a significant decrease in OD570, particularly at 16 µg/mL where OD570 dropped below 0.6, indicating effective biofilm clearance in a dose-dependent manner (Fig. 2A). Statistical analysis confirmed significant differences between the high-concentration groups (8 µg/mL and 16 µg/mL) and the control group (*P < 0.05, **P < 0.01) (Fig. 2A). To further verify the anti-biofilm activity, viable bacterial counts within the biofilm were determined using CFU enumeration. At low concentrations, CFU counts remained largely unchanged. However, a notable reduction was observed at 8 µg/mL and 16 µg/mL (Fig. 2B). Moreover, after normalization to CFU, the biofilm index (OD570/Log_CFU_) in the high-concentration montelukast-treated group remained significantly lower than that of the control group (Fig. S2), indicating that montelukast not only disrupted biofilm structure but also killed bacteria embedded within the biofilm. These findings confirm the potent biofilm-eradicating and bactericidal activity of montelukast at higher concentrations.

### Montelukast effectively treated *S. pneumoniae*-induced sepsis in mice

**Fig 3:**
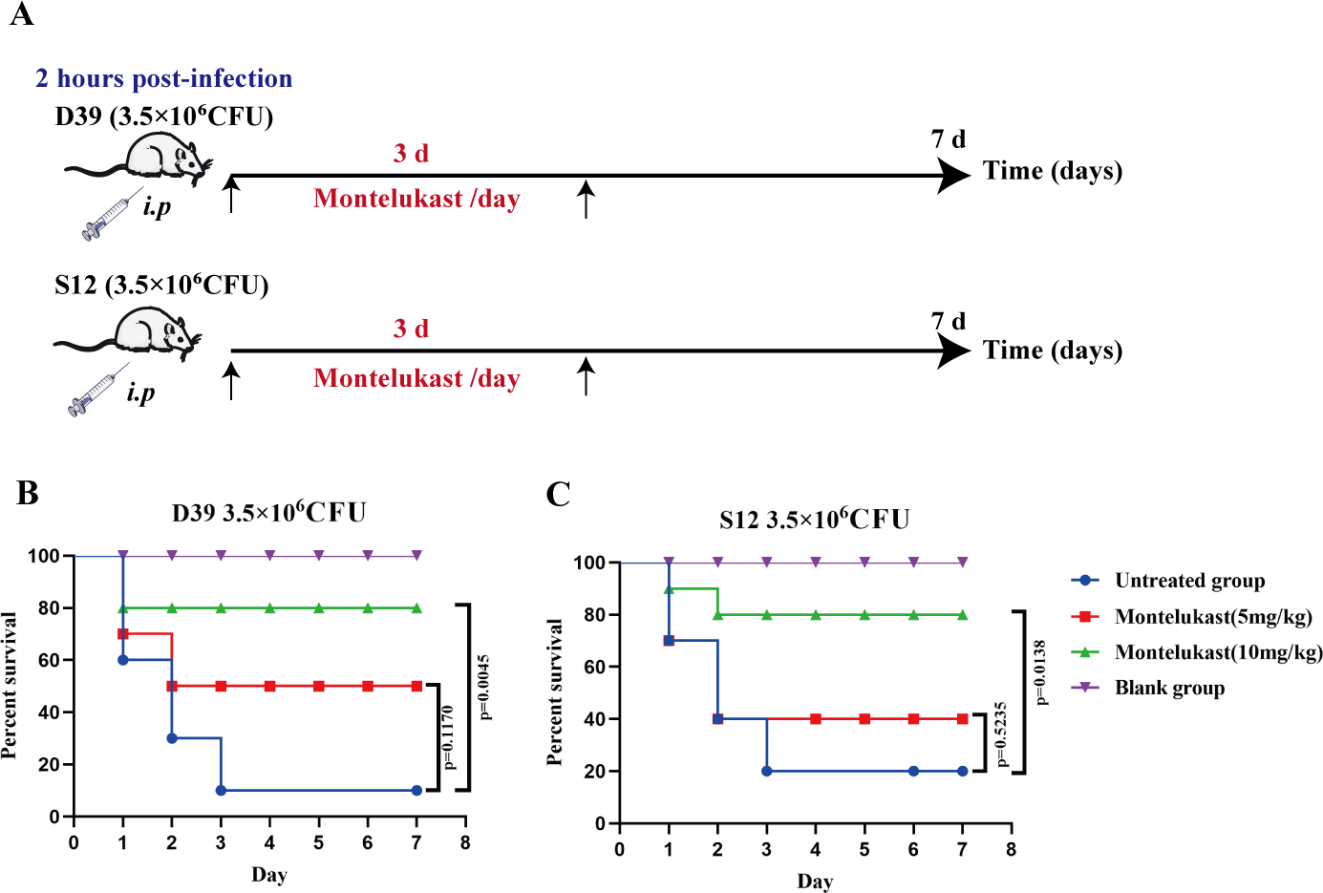
Montelukast was effective in treating lethal sepsis caused by *S. pneumoniae*. (**A**) Schematic diagram of the experimental design. Mice were intraperitoneally infected with *S. pneumoniae* D39 or S12 strain (3.5 × 10⁶ CFU). Montelukast (5 mg/kg or 10 mg/kg) was administered intraperitoneally once daily starting 2 h post-infection for 3 consecutive days. Survival was monitored for 7 days. (**B and C**) Kaplan–Meier survival curves of mice infected with the D39 (**B**) or S12 (**C**) strain. Montelukast treatment significantly improved survival in a dose-dependent manner (*n* = 10 per group, *P* < 0.01, Log-rank test).

**Fig 4:**
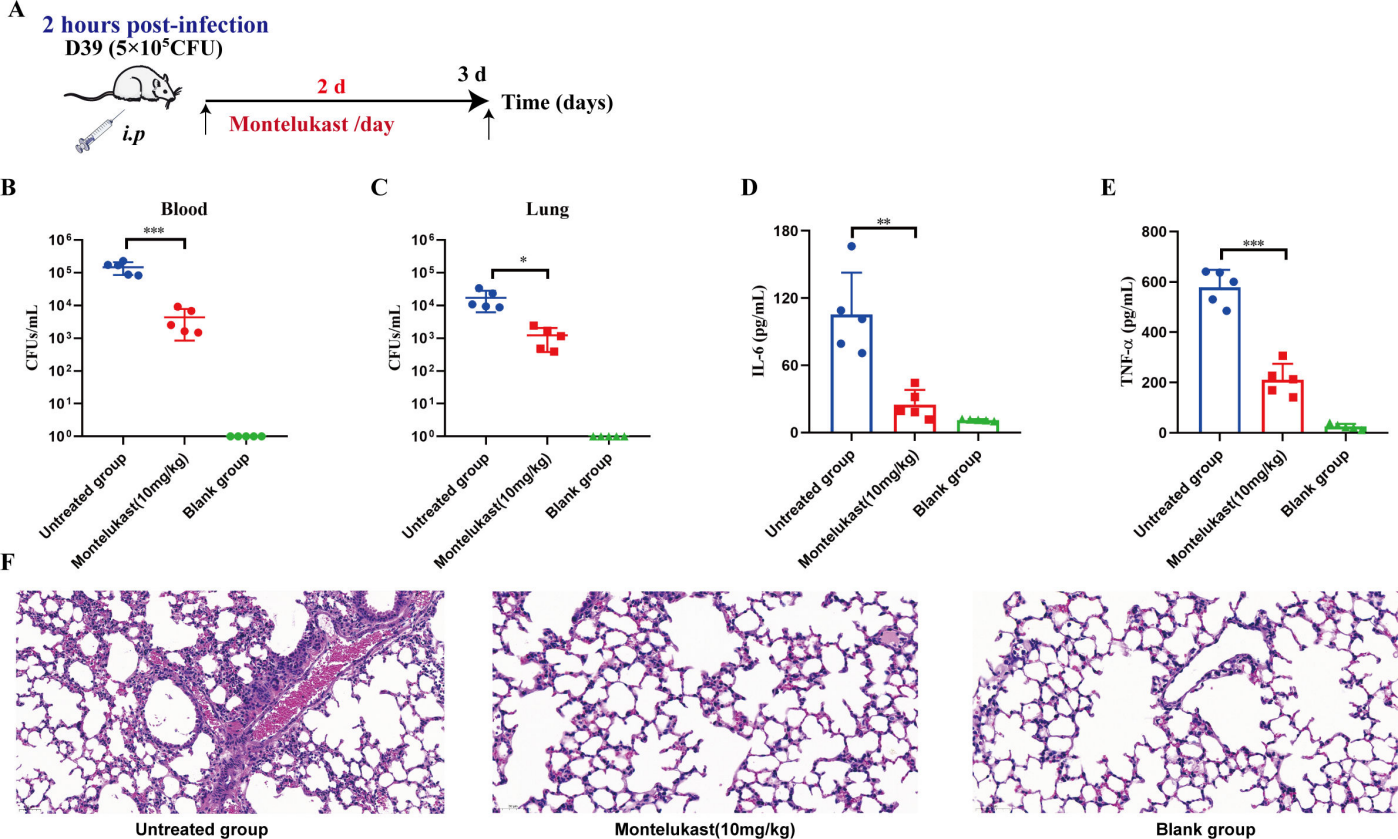
Montelukast was effective in treating non-lethal sepsis caused by *S. pneumoniae*. (**A**) Schematic diagram of the experimental design. A non-lethal sepsis model was established to evaluate the *in vivo* antibacterial and anti-inflammatory activities of montelukast. Bacterial loads in blood (**B**) and lung (**C**) tissues at 72 h post-infection. Montelukast (10 mg/kg) significantly reduced bacterial CFU in both blood and lungs compared with the untreated group (*n* = 5 per group, unpaired *t*-test). Serum cytokine levels of IL-6 (**D**) and TNF-α (**E**) measured by ELISA at 72 h post-infection. Montelukast treatment significantly reduced systemic levels of IL-6 and TNF-α (*n* = 5 per group, unpaired *t*-test). (**F**) Representative histopathological analysis (H&E staining) of lung tissues collected at 72 h post-infection. Scale bar = 50 µm. Data are presented as Mean ± SD, and statistical analysis was performed using unpaired two-tailed *t*-test (*P < 0.05, **P < 0.01, ***P < 0.001).

*S. pneumoniae* is a major cause of sepsis, especially in immunocompromised individuals. The rapid bacterial proliferation and associated inflammatory responses often lead to systemic infection and multiple organ dysfunction (10, 32). Thus, developing therapeutic agents with both antibacterial and anti-inflammatory properties is critical for effective treatment. Having established the *in vitro* antibacterial and anti-biofilm activity of montelukast, we proceeded to assess its *in vivo* efficacy using a mouse sepsis model. As shown in Fig. 3A, mice were intraperitoneally injected with either the D39 reference strain or the multidrug-resistant S12 strain (3.5 × 10⁶ CFU) to induce infection. Two hours post-infection, Montelukast (5 mg/kg or 10 mg/kg) was administered intraperitoneally once daily for 3 consecutive days. Survival was monitored for 7 days. Montelukast treatment significantly improved survival in both the D39 (Fig. 3B) and S12 (Fig. 3C) infection models, with the 10 mg/kg dose yielding the most pronounced effect (*P* < 0.01), increasing survival from 10% to 20% in untreated groups to 80%. In addition to survival analysis, we evaluated bacterial burden and inflammatory cytokine levels. Subsequently, a non-lethal sepsis model was established to evaluate the *in vivo* antibacterial and anti-inflammatory activities of montelukast (Fig. 4A). As shown in Fig. 4B and C, montelukast (10 mg/kg) significantly reduced bacterial load in both blood and lung tissues compared to untreated controls, with blood CFUs decreasing from ~10⁶ to ~10³ (*P* < 0.001) and lung CFUs also showing marked reduction (*P* < 0.05). Serum cytokine analysis revealed that Montelukast reduced IL-6 levels from ~120 pg/mL to ~40 pg/mL (*P* < 0.01) and TNF-α from ~600 pg/mL to ~200 pg/mL (*P* < 0.001), indicating attenuation of systemic inflammation (Fig. 4D and E). Hematoxylin and eosin (H&E) staining of lung tissue further supported these findings, with untreated mice showing alveolar destruction, heavy inflammatory infiltration, and interstitial thickening, whereas montelukast-treated mice exhibited preserved alveolar structure and significantly reduced inflammation, resembling healthy controls (Fig. 4F). In summary, montelukast significantly improves outcomes in *S. pneumoniae*-induced sepsis by reducing bacterial dissemination, suppressing inflammatory cytokine production, and ameliorating tissue damage, highlighting its therapeutic potential through combined antibacterial and anti-inflammatory mechanisms.

### Montelukast disrupted bacterial membrane integrity

**Fig 5:**
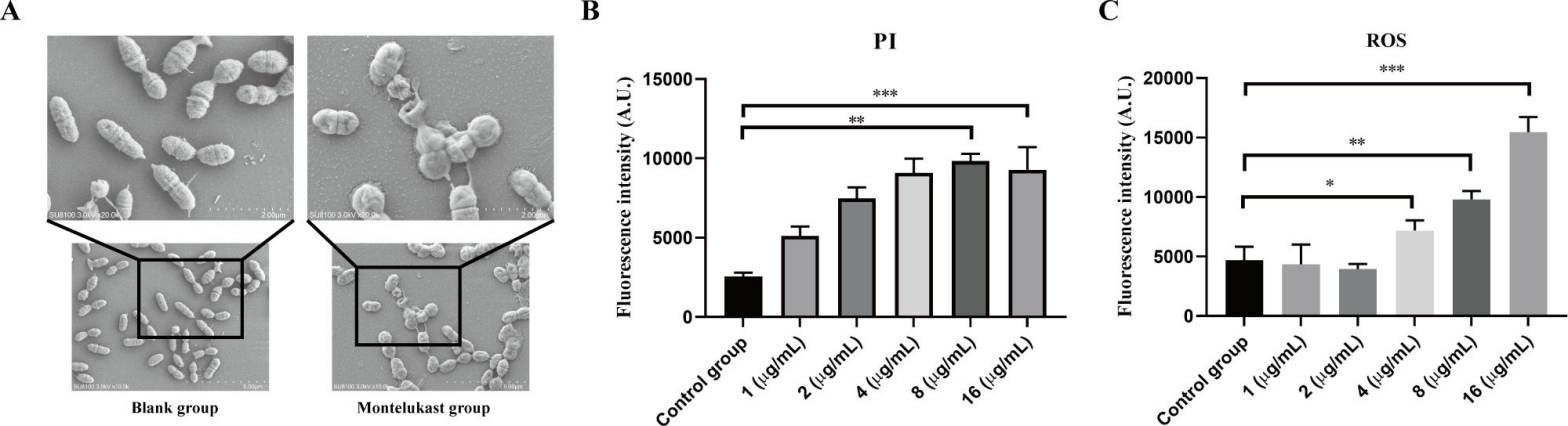
Montelukast disrupted the membrane integrity and induced oxidative stress in *S. pneumoniae*. (**A**) Scanning electron microscopy (SEM) images of *S. pneumoniae* treated with montelukast (16 µg/mL) or untreated (blank group). Montelukast treatment resulted in noticeable morphological alterations, including cell surface wrinkling and membrane disruption. Scale bars = 2 µm (upper), 500 µm (lower). (**B**) PI uptake assay showing increased membrane permeability of *S. pneumoniae* following montelukast treatment at increasing concentrations (1–16 μg/mL). Data are presented as fluorescence intensity (A.U.), indicating increased PI uptake due to compromised membrane integrity. (**C**) Reactive oxygen species (ROS) levels in *S. pneumoniae* after montelukast treatment. Fluorescence intensity increased dose-dependently, indicating elevated intracellular ROS accumulation. Data are presented as Mean ± SD, and statistical analysis was performed using unpaired two-tailed *t*-test (*P < 0.05, **P < 0.01, ***P < 0.001).

**Fig 6:**
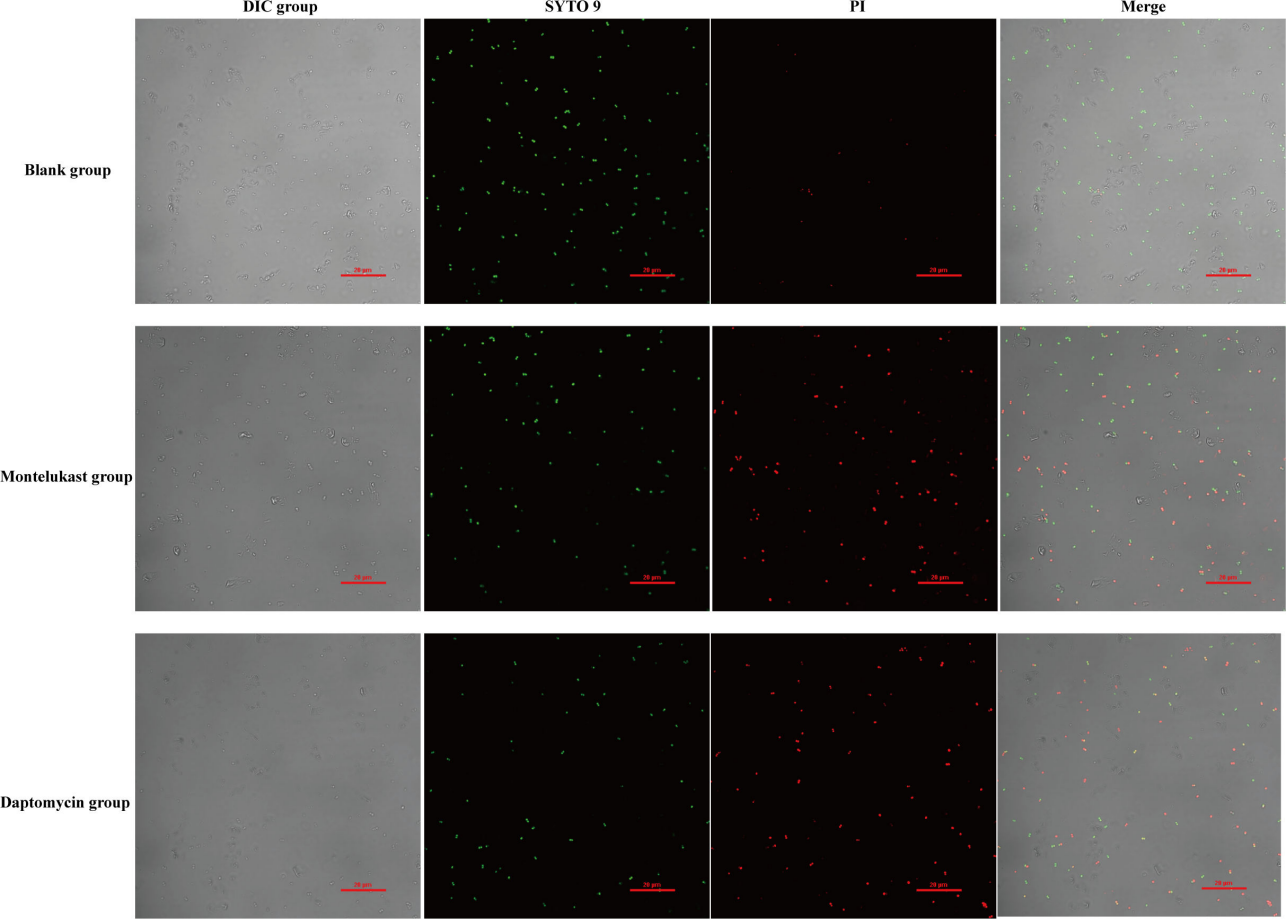
Live/dead bacterial viability staining using SYTO9 and PI. Untreated bacteria in the blank group displayed predominantly green fluorescence, indicating intact membranes. Montelukast-treated (16 µg/mL) and daptomycin-treated groups showed substantial red fluorescence, suggesting membrane damage and bacterial death. The DIC image showed the morphology and distribution of bacteria under bright-field conditions; SYTO 9 stained live bacteria and emitted green fluorescence; PI stained dead bacteria and emitted red fluorescence. The merged image combined the bright-field, SYTO 9, and PI signals to visually distinguish the spatial distribution of live and dead bacteria. Scale bars = 20 µm.

Our previous studies demonstrated that montelukast possessed both bactericidal and anti-biofilm activity against *S. pneumoniae*, with significant therapeutic efficacy in a mouse sepsis model. To further elucidate its mechanism of action, we investigated whether montelukast exerted its antimicrobial effects by disrupting bacterial membrane integrity. Scanning electron microscopy (SEM) images revealed that, compared to the Blank group, *S. pneumoniae* treated with montelukast exhibited distinct morphological alterations, including surface deformation, shrinkage, and membrane rupture (Fig. 5A). These findings suggest that montelukast exerted bactericidal effects by compromising bacterial membrane structure. Then, propidium iodide (PI) staining was performed to assess membrane permeability. The results showed a concentration-dependent increase in PI fluorescence intensity following treatment with montelukast (1–16 µg/mL), indicating a progressive loss of membrane integrity (Fig. 5B). Notably, treatment with 8 µg/mL and 16 µg/mL resulted in significantly higher PI uptake compared to the control group, confirming severe membrane damage (Fig. 5B). And reactive oxygen species (ROS) assays further demonstrated that montelukast induced intracellular oxidative stress in a dose-dependent manner (Fig. 5C). ROS fluorescence intensity markedly increased at 8 µg/mL and 16 µg/mL compared to the control group, suggesting that ROS generation may contribute to its bactericidal mechanism (Fig. 5C). In addition, dual staining with SYTO9 and PI further validated the membrane-disrupting effects of montelukast. In the Blank group, SYTO9-dominated green fluorescence indicated intact cell membranes, whereas montelukast-treated bacteria exhibited strong PI-associated red fluorescence, indicative of extensive membrane damage (Fig. 5D). The extent of membrane disruption was comparable to that observed in the daptomycin (positive control) group, providing additional evidence that montelukast compromised bacterial membrane integrity (Fig. 6D). Consistently, quantitative fluorescence analysis revealed that montelukast treatment resulted in a significant increase in red fluorescence compared to the untreated group (Fig. S1). Collectively, these findings suggested that montelukast exerted its bactericidal effects by damaging the bacterial membrane structure, increasing membrane permeability, and inducing intracellular ROS accumulation. These mechanisms offer crucial support for the therapeutic potential of montelukast as an anti-infective agent.

### Identification of potential molecular target of montelukast

**Fig 7:**
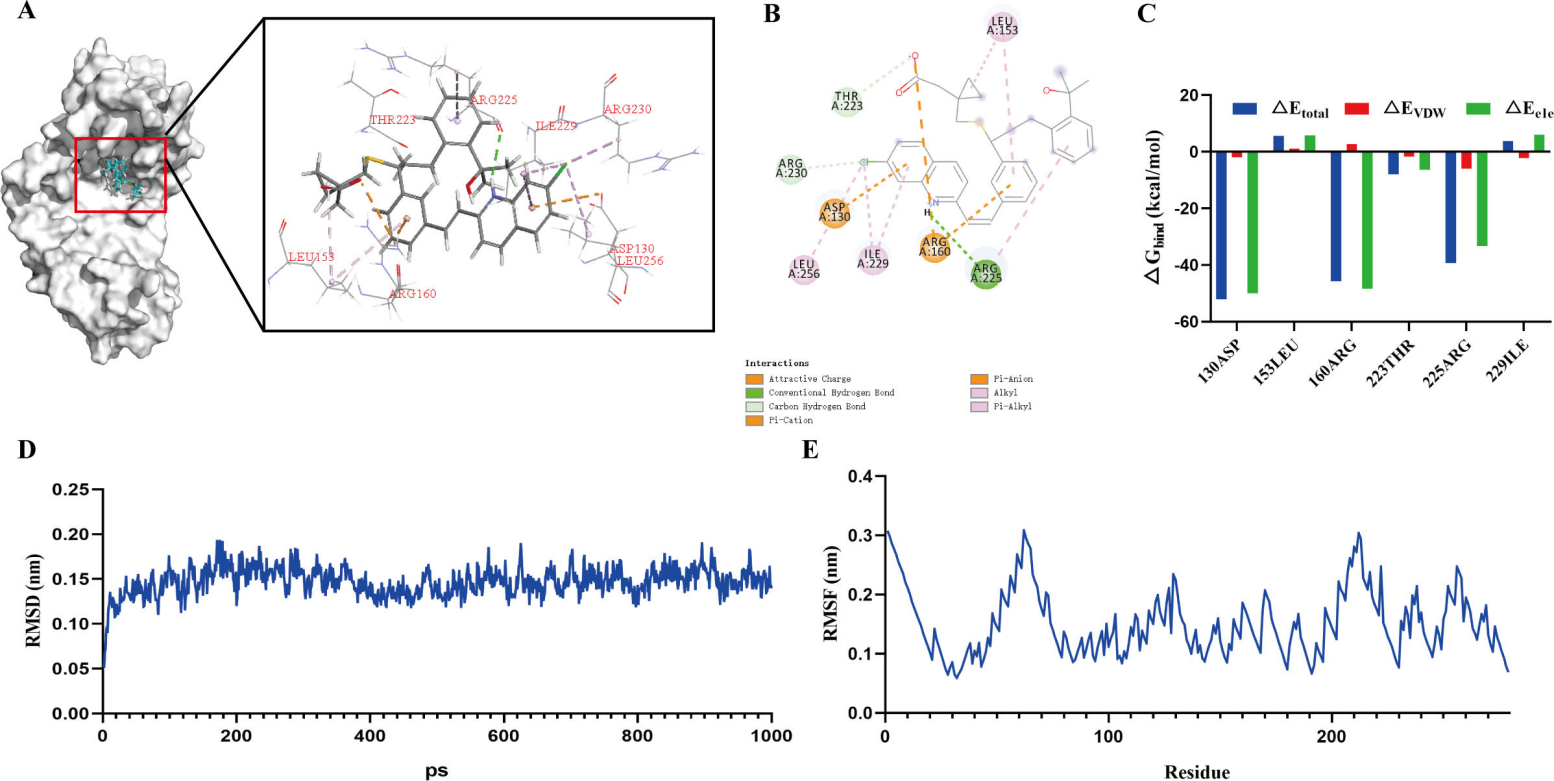
Molecular docking and dynamics simulations revealed potential interaction between montelukast and *S. pneumoniae* pseudouridine synthase. (**A**) Molecular docking model showing montelukast (cyan sticks) bound to the predicted active pocket of *S. pneumoniae* pseudouridine synthase, modeled by AlphaFold2. Enlarged view (right panel) displays the interaction between montelukast and surrounding amino acid residues, including hydrogen bonding and hydrophobic contacts. (**B**) 2D interaction diagram depicting key interactions between montelukast and residues such as ASP130, ARG160, ARG225, ARG230, THR223, LEU153, LEU256, and ILE229. Various interaction types, including hydrogen bonds, π–π interactions, and electrostatic interactions, are annotated. (**C**) Binding free energy decomposition analysis of the montelukast–pseudouridine synthase complex, highlighting the energetic contributions (ΔE_total, ΔE_VDW, ΔE_ele) of key amino acid residues to the overall binding affinity. (**D**) RMSD of the protein-ligand complex over a 1,000 ps molecular dynamics simulation, indicating structural stability. (**E**) RMSF of amino acid residues, reflecting the flexibility and dynamic behavior of the binding site residues.

To systematically elucidate the antibacterial mechanism of montelukast in the treatment of *S. pneumoniae*-induced sepsis, we investigated both its membrane-disrupting activity and potential molecular targets. Previous findings confirmed that montelukast exerted its bactericidal effects by compromising bacterial membrane integrity. Building on this, we explored potential molecular targets of montelukast using *in silico* approaches. Target prediction via the SwissTargetPrediction database identified pseudouridine synthase as a potential binding protein of montelukast. This enzyme plays a critical role in the pseudouridylation of bacterial rRNA and tRNA, which is essential for maintaining ribosome structural stability and translational activity (33). As the crystal structure of *S. pneumoniae* pseudouridine synthase has not yet been resolved, we utilized AlphaFold2 to construct a three-dimensional structural model of the enzyme (Fig. 7A). Molecular docking and dynamic simulations were then performed with montelukast as the ligand. As shown in Fig. 7A, montelukast docked into the core binding pocket of the predicted structure, forming stable interactions through hydrogen bonding, hydrophobic interactions, and π–π stacking with key residues including ASP130, ARG160, ARG225, ARG230, and LEU153. A two-dimensional interaction map further illustrated the nature of these interactions, highlighting conventional hydrogen bonds, alkyl/π-alkyl hydrophobic contacts, and electrostatic attractions (Fig. 7B). These findings elucidated the binding pattern of the ligand–target complex. Then, MM/PBSA free energy decomposition analysis of dynamic simulations indicated that the aforementioned residues, particularly ASP130, ARG160, and ARG225, made significant energetic contributions to complex stability (Fig. 7C). Root mean square deviation (RMSD) analysis also demonstrated that the overall structure of the complex remained stable throughout a 1,000 ps molecular dynamics simulation, with RMSD fluctuations maintained around 0.15 nm (Fig. 7D). Similarly, root mean square fluctuation (RMSF) analysis showed low residue flexibility in most regions, indicating conformational stability of the protein–ligand complex (Fig. 7E). In summary, structural prediction and molecular simulation suggested that montelukast binds to pseudouridine synthase and potentially interferes with its function in ribosome maintenance and protein translation, thereby contributing to its antimicrobial activity. These findings provided theoretical and molecular-level support for the role of pseudouridine synthase as a putative antibacterial target of montelukast.

## DISCUSSION

In recent years, the widespread dissemination of MDR *S. pneumoniae* has led to a progressive decline in the efficacy of conventional antibiotic therapies, highlighting the urgent need to develop novel therapeutic strategies with alternative mechanisms of action (20). This study systematically elucidates the multifaceted pharmacological activities of montelukast in *S. pneumoniae*-induced sepsis, demonstrating its potential as a candidate drug with *in vitro* antimicrobial activity, anti-biofilm properties, and *in vivo* anti-infective and anti-inflammatory effects. These findings not only expand the pharmacological profile of montelukast but also offer a promising solution to the clinical challenge posed by escalating antibiotic resistance. Meanwhile, with the backdrop of rising resistance rates and limited treatment options for *S. pneumoniae* infections, this study introduces a non-antibiotic intervention strategy through drug repurposing, employing a multi-dimensional experimental approach both *in vitro* and *in vivo*. This work broadens the scope of old drug reutilization in the context of infectious diseases.

Montelukast exhibited potent *in vitro* antibacterial activity against *S. pneumoniae*, including clinical isolates with confirmed multidrug-resistant phenotypes. Notably, it demonstrated strong bactericidal effects. Unlike traditional penicillins or macrolides, which primarily target cell wall synthesis or nucleic acid metabolism (34, 35), montelukast exerted its antimicrobial effect through disruption of bacterial membrane structure and increased membrane permeability, ultimately leading to cell lysis and death. The observed membrane damage and elevated intracellular ROS levels are likely interrelated. Disruption of the bacterial membrane compromises cellular homeostasis, leading to increased permeability and oxidative stress. Conversely, excessive ROS accumulation can induce lipid peroxidation and protein oxidation, further exacerbating membrane integrity loss (36, 37). Thus, the increased ROS levels observed following montelukast treatment may act as both a consequence and a driver of membrane disruption, contributing to bacterial cell death. This membrane-targeting mechanism suggested that montelukast functioned as a membrane-disruptive antimicrobial agent. Scanning electron microscopy further confirmed the direct membrane-disrupting effects of montelukast, underscoring the novelty and uniqueness of its antibacterial mechanism. Importantly, membrane-targeting mechanisms are less prone to resistance development compared to traditional molecular targets (38), thereby offering montelukast a distinct advantage in treating resistant bacterial infections and highlighting its strong clinical translational potential. A recent study on *Staphylococcus aureus* has also shown that montelukast interfered with bacterial membrane stability (26), consistent with the findings of this study, and further supporting its potential as a membrane-targeted antimicrobial agent. Moreover, montelukast has been reported to inhibit MDR-related efflux pump systems, significantly enhancing the susceptibility of multidrug-resistant *S. aureus* to various antibiotics (26). We also found in the antibacterial test that it has a synergistic antibacterial effect with some clinical antibiotics against drug-resistant *S. pneumoniae*. This suggested that montelukast could be used not only as a direct bactericidal agent but also as an adjuvant to potentiate the efficacy of existing antibiotics. The present study builds upon these findings by demonstrating that montelukast exerted direct antimicrobial effects against *S. pneumoniae*, and that this activity was mediated through bacterial membrane disruption, rather than solely via efflux pump inhibition. This mechanism complemented existing research and highlights the broad-spectrum, multi-target anti-infective potential of montelukast, particularly in addressing treatment failures associated with antibiotic resistance.

It is noteworthy that with the rapid advances in protein structure prediction and virtual screening technologies in recent years (39, 40), emerging studies have preliminarily identified montelukast as a potential direct antibacterial agent through its interaction with specific bacterial protein targets. Pseudouridine synthase (*Ψ* synthase), a critical ribosomal RNA-modifying enzyme in bacteria, is responsible for the formation of *Ψ* modification sites within rRNA (41). This enzyme plays a vital role in maintaining the structural integrity of ribosomes and ensuring efficient translation (33). In the present study, molecular docking and structure prediction analyses suggested that montelukast may bind to the active site of *Ψ* synthase, thereby blocking its pseudouridylation activity on rRNA. This disruption could lead to ribosomal instability and abnormal protein synthesis, ultimately resulting in bacterial cell death. This potential mechanism provided a novel perspective to explain montelukast’s broad-spectrum antimicrobial activity, indicating that its bactericidal effect is not limited to membrane disruption or metabolic interference but may also involve dysfunction of ribosomal machinery, a deeper intracellular target. This finding aligns well with the current direction of bacterial drug target innovation. Moreover, the inhibition of key bacterial metabolic enzymes through non-traditional allosteric sites has emerged as a promising antimicrobial strategy (29). Previous study have proposed that montelukast may interfere with metabolic regulation by targeting allosteric sites in the glycolytic pathway, leading to metabolic imbalance and accelerated bacterial death (29). Our study extended this concept to translation-associated enzymes by identifying *Ψ* synthase as a potential target, suggesting that montelukast possessed the capacity to bind conserved ribosomal-modifying enzymes and impair their function. This laid a theoretical foundation for the development of novel antimicrobials with low resistance risk and high target selectivity. Particularly in the current context where many resistant bacterial strains exhibit broad resistance to traditional ribosome-targeting antibiotics such as tetracyclines and aminoglycosides, montelukast’s potential targeting of an “upstream” modification step in ribosome maturation may confer broader antimicrobial coverage and a lower propensity for resistance development.

Beyond its activity against Gram-positive bacteria, this study also demonstrated that montelukast significantly disrupts established *S. pneumoniae* biofilms and effectively eradicates biofilm-embedded bacteria. This property held clinical importance for pulmonary infections, otitis media, and invasive bloodstream infections. Biofilms confer bacterial tolerance to antibiotics and immune clearance and are key contributors to chronic and refractory infections. Prior research has shown that montelukast could interfere with *the Pseudomonas aeruginosa* Pqs quorum sensing system, inhibiting both biofilm formation and virulence (27). In our study, we found that montelukast similarly disrupted mature biofilm structures in *S. pneumoniae* and significantly reduced the viability of biofilm-associated bacteria. This cross-species antibiofilm activity suggested that montelukast may target conserved membrane structures or signaling pathways rather than species-specific elements, demonstrating its potential as a broad-spectrum anti-infective agent. Unlike conventional antibiotics that are typically effective only against planktonic bacteria (42), montelukast overcame the treatment limitations posed by biofilm-associated bacterial communities. This feature made it particularly suited for managing chronic or device-associated infections.

In a mouse model of *S. pneumoniae*-induced sepsis, montelukast exhibited marked anti-infective efficacy. Treatment significantly reduced bacterial burdens in peripheral blood and organs, alleviated inflammatory damage and structural destruction in tissues, and prolonged animal survival. Furthermore, montelukast treatment substantially decreased serum levels of TNF-α and IL-6 following infection, thereby ameliorating infection-associated organ dysfunction caused by excessive inflammation. Notably, this anti-inflammatory effect did not result from broad immunosuppression but reflects a balanced modulation of inflammatory pathways upon effective infection control. This helped interrupt the vicious cycle between early infection-induced immune activation and subsequent inflammatory storms, ultimately reducing the risk of organ failure. In contrast to traditional antibiotics that primarily exert bacteriostatic or bactericidal effects through single mechanisms (43), montelukast demonstrated dual antibacterial and anti-inflammatory activities. This dual functionality offers a more promising strategy for the treatment of complex infections such as sepsis. Recent evidence suggested that late-stage sepsis cannot be effectively managed by antimicrobial therapy alone, and anti-inflammatory treatment without effective infection control may increase the risk of secondary infections (44–46). Therefore, agents like montelukast, which combine both mechanisms, were of critical clinical importance. The present study directly addresses this unmet need.

In summary, this study systematically evaluated the multifaceted roles of montelukast in *S. pneumoniae* infections through a comprehensive approach encompassing *in vitro* bacterial growth, biofilm formation, *in vivo* infection models, histopathological analysis, inflammatory cytokine profiling, and mechanistic investigation. The resulting evidence chain provided robust support for the clinical potential of montelukast. Unlike currently known anti-inflammatory drugs that act solely at the host-response level, montelukast directly targets the pathogen and maintains its efficacy *in vivo*, offering a solid mechanistic basis and application scenario for drug repurposing. Particularly in the context of escalating antibiotic resistance, there is an urgent need for candidate agents with novel targets, anti-biofilm properties, and synergistic potential with existing antibiotics. Montelukast clearly represents a feasible and promising option for such next-generation anti-infective strategies.

## MATERIALS AND METHODS

### Bacterial strains and reagents

This study utilized two standard strains of *S. pneumoniae* (NCTC 7466 and ATCC 49619), along with 11 clinical isolates obtained and preserved in our laboratory (strain IDs: S12, S65, S63, S42, S28, S23, S44, S32, S13, S17). All strains were stored in glycerol cryovials at −80  °C. Prior to experiments, strains were revived by inoculation onto TS agar supplemented with 5% fetal bovine serum (FBS) and incubated at 37  °C under 5% CO₂ for 12–18 h. Single colonies were then inoculated into tryptic soy broth (TSB) containing 5% FBS and cultured under the same conditions until reaching the exponential growth phase for subsequent assays.

Montelukast sodium (purity >98%) was purchased from Sigma-Aldrich (USA) and dissolved in DMSO to prepare a 10 mg/mL stock solution, stored at −20  °C. Control antibiotics included penicillin G (PCN), levofloxacin (LEV), clindamycin (CLI), azithromycin (AZM), tetracycline (TET), vancomycin (VAN), and linezolid (LIN), all obtained from MedChemExpress (China) and prepared according to CLSI-recommended concentration standards. All reagents used were of analytical grade or higher.

### Drug susceptibility testing

The minimum inhibitory concentrations (MICs) of montelukast and comparator antibiotics were determined using the broth microdilution method (47). Briefly, bacterial suspensions were adjusted to ~5 × 10⁵ CFU/mL and inoculated into 96-well plates containing serial dilutions of montelukast (final concentration range: 0.125–128 µg/mL). Positive controls (no drug), negative controls (no bacteria), and solvent controls were included. Plates were incubated at 37 °C under 5% CO₂ for 24 h. The MIC was defined as the lowest drug concentration at which no visible bacterial growth was observed. Each strain was tested in triplicate.

### Time-Kill kinetics assay

The methods were based on references with minor modifications (48). A representative multidrug-resistant strain, S12, was selected for time-kill studies. Montelukast was tested at concentrations ranging from 1 to 16 µg/mL. The initial bacterial inoculum was adjusted to 5 × 10⁵ CFU/mL and cultured in TSB supplemented with 5% FBS. OD₆₀₀ values were recorded every 2 h over a 24 h period to monitor growth dynamics. Simultaneously, aliquots were collected at 0, 2, 4, 6, 8, 12, 14, and 16 h, serially diluted 10-fold, and plated on TSA containing 5% FBS. After incubation at 37°C for 24 h, colony-forming units (CFU/mL) were enumerated and time-kill curves were plotted.

### Checkerboard assay

The synergistic effects of montelukast sodium in combination with clinical antibiotics against *S. pneumoniae* were evaluated using the checkerboard broth microdilution method (49). Four clinical isolates (S12, S65, S28, and S44) were cultured in TSB supplemented with 5% FBS at 37 °C to the logarithmic growth phase. Bacterial suspensions were adjusted to a final inoculum of approximately 5 × 10⁵ CFU/mL for use in 96-well plates. Two-dimensional serial dilutions were prepared for montelukast sodium and five antibiotics: levofloxacin (LEV), penicillin (PCN), clindamycin (CLI), azithromycin (AZM), and tetracycline (TET). Each drug was initially prepared at 4 × its MIC, followed by twofold serial dilutions across the wells. Equal volumes of the two drugs were combined in the wells, along with the bacterial inoculum, resulting in a final volume of 200 µL per well. Plates were incubated at 37°C under 5% CO₂ for 24  h. The minimum inhibitory concentration (MIC) was recorded for each drug alone and in combination. The fractional inhibitory concentration index (FICI) was calculated using the formula: FICI = MIC_A alone_/MIC_A in combination_ + MIC_B alone_/MIC_B in combination_. Where drug A was montelukast and drug B was the corresponding antibiotic. Synergy was defined as FICI ≤ 0.5, partial synergy as 0.5 < FICI ≤ 1, no interaction as 1 < FICI ≤ 4, and antagonism as FICI > 4.

### Biofilm disruption assay

The crystal violet staining method was used to evaluate the biofilm clearance activity of montelukast against *S. pneumoniae* S12 (50). Bacterial suspensions were adjusted to 1 × 10⁷ CFU/mL and inoculated into 96-well plates (200 µL/well) with TSB containing 5% FBS and varying concentrations of montelukast (0.5–8 µg/ml). After 48 h of static incubation, the supernatant was removed, and wells were gently washed three times with PBS to remove planktonic bacteria. Biofilms were stained with 200 µL of 0.1% crystal violet for 20 min and then washed and destained with 200 µL of 95% ethanol. Absorbance was measured at 570 nm (OD₅₇₀). Each treatment group included six technical replicates, and the experiment was independently repeated three times. Following PBS washing (prior to staining), biofilms were disrupted using a combination of ultrasonic treatment (40 kHz, 5 min) and repeated pipetting. The resulting suspension was serially diluted and plated on TSA for CFU counting after 24 h of incubation at 37  °C.

### Mouse sepsis model and *in vivo* therapeutic evaluation

The experimental methods were followed with minor modifications (51). Specific pathogen-free (SPF) C57BL/6 mice (6–8 weeks old, 18–22 g, equal numbers of males and females) were purchased from GemPharmatech Co., Ltd. and acclimatized for one week prior to experimentation. *S. pneumoniae* strains D39 or S12 were cultured to logarithmic phase, harvested by centrifugation, washed, and resuspended in sterile PBS to a final concentration of 3.5 × 10⁶ CFU/200 µL. Mice were intraperitoneally injected to establish a sepsis model. Two hours post-infection, mice were randomly assigned to treatment groups receiving intraperitoneal montelukast (5 mg/kg or 10 mg/kg) once daily for three consecutive days. Control groups received an equal volume of vehicle. Survival was monitored for seven days, and Kaplan–Meier survival curves were plotted.

To assess bacterial burden and anti-inflammatory effects, mice (*n* = 5 per group) were intraperitoneally inoculated with 5 × 10⁵ CFU/200 µL of bacterial suspension. Two hours post-infection, montelukast was administered as described above (10 mg/kg, once daily for 2 days). On day 3 post-infection, mice were anesthetized, and blood was collected from the heart for measurement of serum IL-6 and TNF-α levels using commercial ELISA kits. Lung tissues were homogenized, serially diluted, and plated on TSA supplemented with 5% fetal bovine serum for bacterial enumeration after overnight incubation at 37°C. Portions of lung tissues were fixed in 4% paraformaldehyde for histopathological analysis.

### Scanning electron microscopy for morphological analysis

*S. pneumoniae* ATCC 49619 was cultured overnight and subcultured to mid-log phase. Bacterial cells were washed and resuspended in PBS to an OD600 of 0.5. Montelukast at 4 × MIC was added and incubated for 2 h. Cells were then washed three times with PBS and fixed in electron microscopy fixative (Servicebio) for 2 h at room temperature, followed by overnight fixation at 4°C. After washing three times with PBS (15 min each), samples were transferred to 1% osmium tetroxide (OsO₄, Ted Pella Inc.) and incubated for 2 h at room temperature. Subsequently, the samples were dehydrated through a graded ethanol and isoamyl acetate series. Samples were dried using a critical point dryer (K850, Quorum, Laughton, East Sussex, UK), mounted on aluminum stubs with carbon adhesive tape, and sputter-coated with gold for 30 s. Images were captured using a scanning electron microscope (SU8100, HITACHI, Tokyo, Japan).

### Assessment of membrane integrity and ROS generation

PI staining was used to assess bacterial membrane integrity. Bacteria were treated with various concentrations of montelukast and incubated at 37°C for 2 h. After treatment, cells were centrifuged, washed, and stained with PI (5 µg/mL) for 15 min, followed by PBS washing to remove excess dye. A 200 µL aliquot of each suspension was transferred to a black 96-well plate, and fluorescence intensity was measured using a multimode plate reader (excitation: 535 nm; emission: 615 nm) (52). ROS levels were detected using the fluorescent probe DCFH-DA (10 µM). Bacterial suspensions were incubated with DCFH-DA at 37°C for 30 min in the dark. After washing twice with PBS and resuspension, bacteria were transferred to a black 96-well plate containing different concentrations of montelukast. After 2 h incubation, fluorescence was recorded at Ex492 nm/Em525 nm (53).

### LIVE/DEAD membrane integrity assay

Membrane damage was further evaluated using the LIVE/DEAD BacLight Bacterial Viability Kit (Thermo Fisher) (54, 55). After treatment with montelukast (4 × MIC) for 2 h, *S. pneumoniae* ATCC 49,619 cells (OD600 = 0.5) were washed and resuspended in PBS. A mixture of SYTO 9 (5 µM) and PI (1 µM) was added to each well (500 µL/well) and incubated at room temperature for 30 min in the dark. Bacterial viability was observed and imaged under a laser scanning confocal microscope. Live bacteria stained green with SYTO 9, while dead bacteria stained red with PI. Daptomycin at 16 µg/mL was used as a positive control. Quantitative fluorescence analysis was done by ImageJ software.

### Molecular docking and molecular dynamics simulation

Potential targets of montelukast were predicted using SwissTargetPrediction (56), identifying pseudouridine synthase as a candidate. The 3D structure was modeled using AlphaFold2. The 2D structure of montelukast was obtained from the PubChem database (http://pubchem.ncbi.nlm.nih.gov/), converted to a 3D structure in ChemOffice, and saved as a mol2 file. Molecular docking was performed using AutoDock Vina 1.5.6 to explore protein-ligand interactions. Protein and ligand structures were pre-processed by adding hydrogens, removing water molecules, and defining rotatable bonds. Docking grid parameters were set, and the optimal binding conformation was selected based on binding affinity scores. The interaction between the compound and key residues was visualized in 2D and 3D using Discovery Studio 2019.

Molecular dynamics (MD) simulations were conducted using Gromacs 2023 for 1000 ps (57). The CHARMM36 force field was applied for the protein, and GAFF2 was used for ligand topology (58). The protein-ligand complex was placed in a cubic box with periodic boundary conditions, filled with TIP3P water molecules. Long-range electrostatics were calculated using the particle mesh Ewald (PME) method and the Verlet cutoff scheme. Energy minimization and equilibration were carried out using NVT and NPT ensembles (100,000 steps each) with coupling constants of 0.1 ps. Lennard-Jones and Coulombic interactions were truncated at 1.0 nm. Final MD simulations were performed for 1,000 ps at 300 K and 1 atm.

### Statistical analysis

All data were analyzed using GraphPad Prism 8.0. Statistical comparisons were conducted using unpaired two-tailed Student’s *t*-tests or one-way ANOVA. Data are expressed as mean ± standard deviation (Mean ± SD). Differences were considered statistically significant at *P < 0.05, highly significant at **P < 0.01, and extremely significant at ***P < 0.001.

## SUPPLEMENTAL MATERIAL

Figures S1 and S2 spectrum.01221-25-s0001.docx Fig. S1: Biofilm formation index (OD₅₇₀/LogCFU) of *S. pneumoniae* after treatment with different concentrations of montelukast. Fig. S2: Percentage of Syto Green (green) and PI (red)-labeled *S. pneumoniae*.

ASM does not own the copyrights to Supplemental Material that may be linked to, or accessed through, an article. The authors have granted ASM a non-exclusive, world-wide license to publish the Supplemental Material files. Please contact the corresponding author directly for reuse.
