# Supplementary material for: Montelukast treats Streptococcus pneumoniae-induced sepsis via antibacterial and anti-inflammatory activities
Source: Microbiol Spectr. 2025 Oct 15;13(11):e01221-25. doi: 10.1128/spectrum.01221-25 (PMC12584749; doi:10.1128/spectrum.01221-25)
Supplement: Figures S1 and S2 — Fig. S1: Biofilm formation index (OD₅₇₀/LogCFU) of S. pneumoniae after treatment with different concentrations of montelukast. Fig. S2: Percentage of Syto Green (green) and PI (red)-labeled S. pneumoniae. [file spectrum.01221-25-s0001.docx]

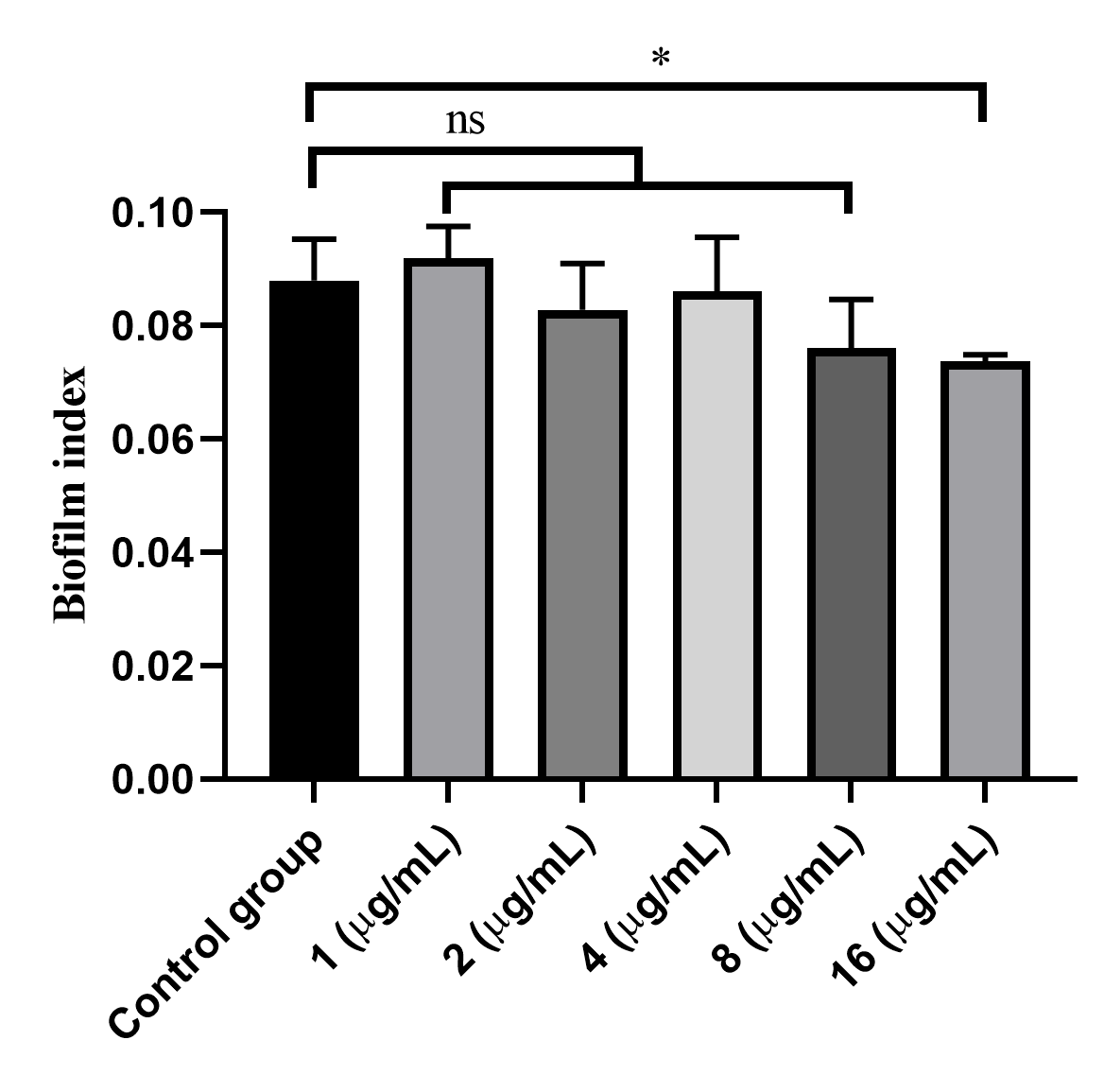


**Figure. S1.** Biofilm formation index (OD₅₇₀/Log_CFU_) of *S. pneumoniae* after treatment with different concentrations of montelukast. Bacteria were incubated with montelukast at indicated concentrations, and biofilm biomass was quantified using crystal violet staining. Data are presented as Mean ± SD, and statistical analysis was performed using unpaired two-tailed t-test (*P < 0.05)


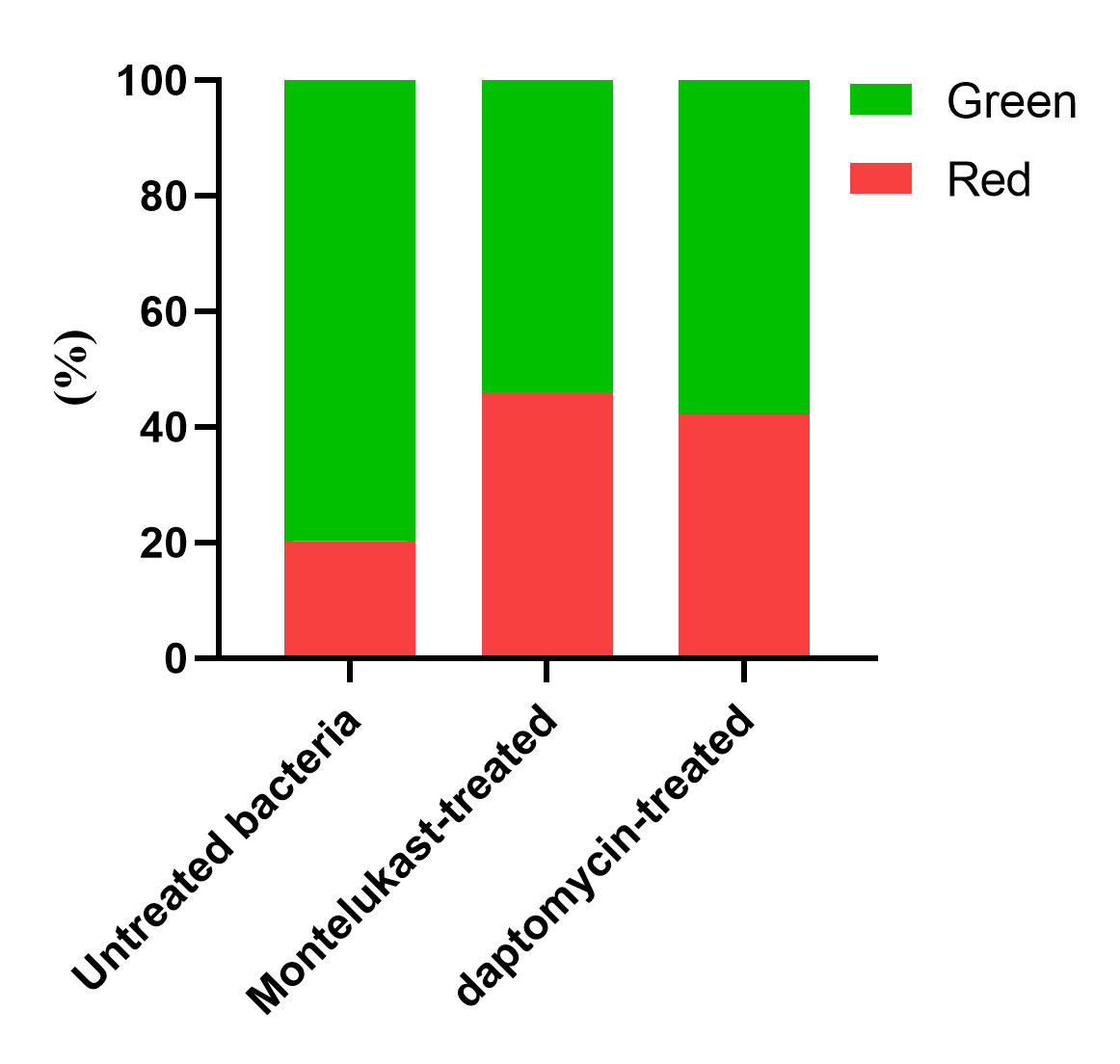


**Figure. S2.** Percentage of Syto Green (green) and PI (red)-labeled *S. pneumoniae*.
